# Long noncoding RNA CCAT2 reduces chemosensitivity to 5‐fluorouracil in breast cancer cells by activating the mTOR axis

**DOI:** 10.1111/jcmm.17041

**Published:** 2022-02-15

**Authors:** Daoping Zhou, Juan Gu, Yueping Wang, Bing Luo, Mei Feng, Xuedong Wang

**Affiliations:** ^1^ Department of Medical Laboratory Science Anhui No.2 Provincial People’s Hospital Hefei Anhui China; ^2^ Department of Oncology Anhui No.2 Provincial People’s Hospital Hefei Anhui China; ^3^ Department of Medical Laboratory Science The Fifth People’s Hospital of Wuxi Nanjing Medical University Wuxi Jiangsu China; ^4^ Department of Pathology The Fifth People’s Hospital of Wuxi The Medical School of Jiangnan University Wuxi Jiangsu China; ^5^ Department of Biology College of Arts & Science Massachusetts University Boston Massachusetts USA

**Keywords:** 5‐Fu, Breast cancer, CCAT2, Drug resistance, mTOR pathway

## Abstract

Breast cancer (BC) is the most prevalent cancer in women and the second leading cause for cancer‐related death in women. LncRNA CCAT2 is involved in BC cell drug sensitivity. Drug resistance of BC cells after chemotherapy is the main obstacle to therapeutic effects. This study explored whether BC cell drug sensitivity to 5‐Fu was related to lncRNA CCAT2‐regulated mTOR pathway. Normal breast tissues and BC tissues before/after neoadjuvant chemotherapy were collected, and CCAT2 expression was detected by RT‐qPCR. Correlation between CCATA2 expression and neoadjuvant chemotherapy efficacy was analysed using the Kendall's tau‐b correlation analysis. Normal breast epithelial cells and BC cell lines were cultured. BC cell lines were treated with 5‐Fu, and CCAT2 mRNA level in cells was detected. The 5‐Fu‐resistant MCF‐7/5‐Fu and MDA‐MB‐231/5‐Fu cells were treated with CCAT2 overexpression/knockdown or CCI‐779 (the mTOR pathway inhibitor). The mTOR pathway levels were detected. Expression of apoptosis‐related factors was identified. A subcutaneous xenograft model was carried out. High CCAT2 expression was detected in BC tissues and BC drug‐resistant cells after neoadjuvant chemotherapy, and a negative link was revealed between CCAT2 expression and efficacy of neoadjuvant chemotherapy. p‐mTOR/mTOR in 5‐Fu‐resistant BC cells with inhibited CCAT2 was decreased, while CCAT2 overexpression activated the mTOR pathway. IC50 value, proliferation, cells in S phase increased and apoptosis reduced after CCAT2 overexpression. After si‐CCAT2 or CCI‐779 treatment, the growth rate of transplanted tumours was inhibited, while promoted after CCAT2 overexpression. CCAT2 may reduce BC cell chemosensitivity to 5‐Fu by activating the mTOR pathway.

## 1 INTRODUCTION

Breast cancer (BC) is the most common cancer in females with approximately 400,000 deaths per year worldwide.^1^, ^2^, ^3^ As a severe public health problem in developed countries, it has also become a growingly important issue in developing countries, where the incidence of BC is growing at a rate of 5% per year.^4^ Women of all ages are at risk of developing BC, and more than 90 percent of patients are possible to be cured through effective comprehensive treatment once diagnosed at early stage.^5^ There are many established risk factors for BC, including aging, early menarche, family history, elder age at first live childbirth, late menopause, genetic causes, alcohol consumption, history of smoking, and obesity.^6^ Radiotherapy plays a crucial part in BC treatment.^7^ Besides, 5‐fluorouracil (5‐FU)‐based combination chemotherapy is widely used for BC treatment, while recurrence and chemotherapy resistance are major obstacles leading to the high mortality in most patients.^8^ Thereby, it is imperative to know the molecular mechanisms of chemotherapy resistance in BC for improving clinical efficacy.

Long noncoding RNAs (lncRNAs) are usually expressed in a specific manner, which exert specific functions in multiple kinds of diseases and human cancers.^9^ More and more dysregulated lncRNAs are regarded as oncogenes that would promote the development and progression of cancers.^10^ Additionally, the regulatory roles of lncRNAs in drug resistance are studied. For example, Leucci E recently pointed out that lncRNAs are applicable to sensitize cancer cells to multiple kinds of treatments.^11^ Colon cancer‐associated transcript 2 (CCAT2), mapping to 8q24, is upregulated in many tumour tissues and links with clinical characteristics and prognosis in multiple kinds of malignancies, like bladder cancer, gastric cancer, and cervical cancer.^12^, ^13^, ^14^, ^15^ In addition, CCAT2 is also proved to promote BC progression via the Wnt pathway.^16^ The mammalian target of rapamycin (mTOR) is important in the extracellular and intracellular signals.^17^, ^18^ The mTOR pathway is downstream of PI3K/Akt, a well‐known pathway mainly out of regulation in cancer, which is also involved in BC.^19^ Now we tried to discuss the regulatory roles of CCAT2 in BC drug sensitivity to 5‐Fu via the mTOR axis.

## 2 MATERIALS AND METHODS

### 2.1 Study subjects

One hundred BC patients (all females, 26–82 years; average age of 49 years) who were first diagnosed and received neoadjuvant chemotherapy in Anhui No.2 Provincial People's Hospital from January 2014 to January 2018 were collected. Pathological data of all patients were complete and clear, including 79 cases of invasive ductal carcinoma, 11 cases of intraductal carcinoma, 7 of invasive lobular carcinoma, 2 of clear cell carcinoma, and 1 of mucinous adenocarcinoma. All patients underwent radical mastectomy with neoadjuvant chemotherapy after three courses of CMF (cyclophosphamide 600 mg/m^2^ d1, d8, methotrexate 0.4 mg/kg d1, fluorouracil [5‐Fu] 12 mg/kg d1‐d5, 21 days as a cycle). Then, BC samples were harvested by core needle biopsy or excisional biopsy before chemotherapy, and by biopsy and chemotherapy after radical resection. Besides, the neoadjuvant chemotherapy efficacy in patients was assessed based on the unified standard formulated by WHO. In addition, before and after neoadjuvant chemotherapy, BC patients took the physical examination, coordinate mapping, breast B‐mode ultrasound, and molybdenum target to evaluate the efficacy. Also, before chemotherapy, the size of tumours obtained from the excisional biopsy was quantified using B‐mode ultrasonography. The changes of the tumours were followed up, and the efficacy was judged comprehensively by biopsy and pathology after radical resection. Samples were split into: complete remission (CR): no tumour detected using clinical means; partial remission (PR): reduced >50% in breast lumps; stable disease (SD): reduced <50% and increased <25% in breast lumps; progressive disease (PD): increased volume of breast lumps >25%.^20^ CR + PR was the total effective rate. All BC patients were diagnosed for the first time. Besides, 90 normal breast tissues were collected from the healthy volunteers who came to Anhui No.2 Provincial People's Hospital for breast physical examination during the corresponding period. The healthy subjects were all female, aged 28–67 years, with an average age of 48.5 years. There was no significant difference in mean age between the two groups. The experiment got the approval of the ethics committee of Anhui No.2 Provincial People's Hospital. All subjects signed a statement of informed consent.

### 2.2 Cell culture

BC cell lines (MDA‐MB‐231, SKBR‐3, MCF‐7, and HCC‐1937) and normal breast epithelial cells (MCF10) were provided by the cell bank of the Chinese Academy of Sciences. The corresponding 5‐Fu drug‐resistant cell lines were prepared by exposure to 5‐FU. Firstly, BC cells were cultivated in RPMI‐1640 complete medium with 10% FBS at 37°C with 5% CO_2_; afterwards, cells were subcultured when the confluence reached 90%. And then, the cells were added with 5‐Fu to a final concentration of 3.84 mol/L. After 48‐h incubation, cells were subcultured with the renewed medium. In addition, the drug concentration was then increased gradually, and finally, the cell line that tolerated 23 mol/L 5‐Fu was obtained,^21^ namely MCF‐7/5‐Fu, SKBR‐3/5‐Fu, MDA‐MB‐231/5‐Fu, and HCC‐1937/5‐Fu, respectively.

### 2.3 Cell transfection

MCF‐7 cells and MDA‐MB‐231 cells with the greatest difference and minimum difference, respectively, in CCAT2 expression compared with normal breast epithelial cell MCF10 were selected for cell experiment. MCF‐7/5‐Fu cells and MDA‐MB‐231/5‐Fu cells were grouped into blank group, empty plasmid group, si‐CCAT2 group (cells transfected with CCAT2 interference plasmid of), CCAT2 group (cells transfected with CCAT2 overexpression plasmid), negative control (NC) group (cells treated with DMSO), CCI‐779 (an inhibitor of mTOR) group (cells treated with an inhibitor of mTOR which was dissolved with DMSO and, the final concentration was 20 mmol/L), and CCAT2 + CCI‐779 group (cells treated with CCI‐779 [final concentration was 20 mmol/L] and CCAT2 overexpression plasmid). Empty plasmids, siRNA interference plasmids, and overexpression plasmids were provided by Shanghai GenePharma Co. Ltd., (Shanghai, China). Meanwhile, cells were transfected according to Lipofectamine 2000 (Invitrogen, Carlsbad, CA, USA) instructions. Cells were cultured in incubators and subsequently detected 48 h later.

### 2.4 Cell counting kit‐8 (CCK‐8) assay

After 48‐h treatment, cells were collected, digested, and inoculated into the 96‐well plates at 8 × 10^3^/well (200 μl per well). After cell adherence, 5‐Fu at 0.10 μmol/L, 0.50 μmol/L, 2.50 μmol/L, 12.50 μmol/L, 20.00 μmol/L, and 40.00 μmol/L were given to cells, respectively. Three replicates were set in each group at each concentration. Zero‐adjusting well and blank control group were set at the same time. After adding the drug, cells were continuously cultivated at 37°C with 5% CO_2_ and saturated humidity. After discarding the medium and adding 100 μl fresh medium with 10 μl CCK‐8 solution (Shanghai Beyotime Biotechnology Co. Ltd.), the plates were cultivated in the cell incubator for 2 h. Next, OD value at 450 nm wavelength was determined by an enzymatic marker (Biorad, USA). IC50 was evaluated by probit regression analysis of SPSS software.^22^

### 2.5 5‐ethynyl‐2'‐deoxyuridine (EdU) assay

Cells at the logarithmic growth phase were detached with trypsin and triturated into a single cell suspension. After counting, cells were seeded on 96‐well plates at 1 × 10^4^ cells per well. The culture liquid in each well was 200 μl. After 48 h, the cells entered an exponential growth phase. The cells were stained with EdU, and the 100‐μl complete culture medium containing EdU was put into each well for 2‐h incubation. Afterwards, cells were rinsed in PBS twice and cultured in 100 μl 4% polyformaldehyde for 30 min. Next, cells were added with glycine (final concentration was 2 mg/ml) for oscillating on rockers for 5 min, added with 100 μl PBS for 5‐min oscillating, and added with 0.5%Triton X‐100 (100 μl) for 5 min oscillating. After PBS cleaning, 100 μl 1 × Apollo dyeing reaction solution was put into each well, and the dyeing solution was discarded after 30 min shaking. Later, cells were added with 0.5% Triton X‐100 for 10 min shaking, followed by nucleus staining with 100 μl 1 × Hoechest 100 after discarding the 0.5% Triton X‐100. After 30 min shaking, the dyeing reaction solution was removed, and cells were rinsed in PBS, observed under the microscope, and then analysed with Image‐Pro Plus software.

### 2.6 Flow cytometry

AnnexinV/propidiom iodide (PI) double staining method was used for cell apoptosis detection. After 48‐h grouping, cells were harvested and adjusted to 1 × 10^6^/ml. Cell suspension (0.5 ml) was put into centrifugal tubes and added with 1.25 μl Annexin V‐FITC (Nanjing Kaiji Biotechnology Co. Ltd.). After 15‐min reaction in the dark, the cell suspension was centrifuged at 1000 rpm for 5 min with the supernatant removed. Next, cells were suspended with 0.5 ml pre‐cooling combined buffer and added with 10 μl PI for immediate detection on a flow cytometer.

Cell cycle was detected using PI single staining. After grouping for 48 h, cells were fixed with −20°C pre‐cooled 75% ice ethanol overnight at 4°C, then centrifuged, washed with cold PBS, and precipitated twice to remove the immobilization solution. Then cells were added with RNaseA and bathed in water for 30 min. Afterwards, cells were dyed with PI, followed by even mixing. Finally, the red fluorescence was recorded via flow cytometry for cell cycle detection and counting the proportion of G0/G1, S, and G2/M cells.

### 2.7 Subcutaneous xenograft nude mouse model

One hundred and forty BALB/C nude female mice (6 weeks, 16–21 g) provided by Beijing Union Animal Center were reared on a laminar flow shelf without specific pathogen conditions. When MCF‐7/5‐Fu cells grew to the logarithmic growth phase, 140 nude mice were injected subcutaneously with 0.2 ml suspension (5 × 10^6^ cells) via the axilla of the forelimb. The growth of nude mice was observed. Two weeks later, 135 nude mice had axillary nodules of about 100 mm^3^ in volume, which suggested the successful modelling. A total of 100 nude mice were at random assigned into 5 groups, 20 in each group, namely NC group (injected with empty plasmid, 30 μg/200 μl/time), si‐CCAT2 group (injected with siRNA interference plasmid of CCAT2, 30 μg/200 μl/time), CDR1as group (injected with overexpression plasmid of CCAT2, 30 μg/200 μl/time) and CCI‐779 group (injected with mTOR signalling pathway inhibitor at a concentration of 50 mg/kg/time), and CCAT2 + CCI‐779 group (injected with CCAT2 overexpression plasmid and CCI‐779). Intra‐mass injection was carried out once a week for four weeks. All mice were given 5‐Fu (10 mg/kg) by slow injection via the caudal vein to observe the size of tumours and the response of chemotherapy. The injection was performed three consecutive days as a course of treatment and then conducted for a course of treatment after two weeks. The long diameter (a) and short diameter (b) of tumours were gauged before injection with the plasmid or CCI‐779, and tumour volume = ab^2^/2. The growth curve was drawn. The mice were anesthetized by intraperitoneal injection with 1% pentobarbital sodium (30 mg/kg) at the end of the fourth week, and the tumour specimens were removed by surgical resection. The tumour specimens were subjected to pathological sections for immunohistochemistry and tissue homogenate for RT‐PCR and Western blot to detect the corresponding indexes. After tumour removal, the nude mice were killed using the decapitation method.

### 2.8 Immunohistochemistry

Tissue slices of transplanted tumours were taken and added with 30% H_2_O_2_ to block the endogenous enzyme. The slices were heated to boil in antigen retrieval solution. After 5 min of cooling, the slices were reheated and cooled twice. After cooling, slices were added with 5% BSA solution with the excess liquid removed. After that, slices were cultured with rabbit anti‐mouse Ki‐67 primary antibody (ab15580, 1:1000, Abcam) overnight at 4°C and added with biotinylated goat anti‐rabbit IgG (ab6721, Abcam). Then, slices were washed in PBS and developed in DAB (ZSGB‐Bio). The staining results were observed under the microscope. The cells with brown and yellow were regarded as positive expressions. Three slices were taken from each specimen. Five visual fields were selected at random from each slice to calculate the positive rate of Ki‐67.

### 2.9 Reverse transcription quantitative chain reaction (RT‐qPCR)

The clinical sample tissues or cells or homogenate of transplanted tumour tissues were collected, and total RNA was extracted by Trizol total RNA extraction kit. The optical density (OD) values of 5 μl RNA samples at 260 and 280 nm of ultraviolet spectrophotometer were read by diluting 20 times with super‐pure water without RNA enzyme. RNA concentration and purity were determined. OD260/OD280 ratio was within 1.7–2.1, indicating the purity met the needs of subsequent experiments. Real‐time qPCR was performed with ABI7500 qPCR (ABI). The reaction conditions were as follows: 95°C, 10 min and 50 cycles of 95°C, 15 s, 60°C 1 min, 72°C, 40 s. The primers used in the reaction are shown in Table 1. The data were analysed using the 2^−ΔΔCt^ method. GAPDH was used as the internal parameter and normalized. The experiment was repeated three times.

**TABLE 1:** Primer sequences for RT‐qPCR

	Primer sequences
CCAT2	5’‐CCCTGGTCAAATTGCTTAACCT−3’
	5’‐TTATTCGTCCCTCTGTTTTATGGAT−3’
mTOR	5’‐CCACTGTGCGGATCATTTC−3’
	5’‐CTGGATGAGCATCTTGCG−3’
GAPDH	5’‐CCACATCGCTCAGACACCAT−3’
	5’‐ACCAGGCGCCCAATACG−3’

### 2.10 Western blot

The total proteins in cells or the homogenate of transplanted tumour tissues were extracted by protein lysate and then quantified by Bradford method (Thermo Fisher Science). After sodium dodecyl sulphate polyacrylamide gel electrophoresis) electrophoresis in 50 μg protein, the proteins were transferred to the polyvinylidene fluoride membrane (Millipore), which was sealed with 5% skimmed milk at 37°C for 1 h on a shaker. After that, the membrane was then added with mouse anti‐human mTOR (ab2732, 1:1000), p‐mTOR (ab109268, 1:1000), Bcl‐2 (ab32124, 1:1000), Bax (ab32503, 1:1000), Caspase‐3 (ab32503, 1:1000), Cleaved‐Caspase‐3 (ab32042, 1:100), β‐actin (ab8226, 1:1000) primary antibodies for incubation at 4°C overnight. After that, the membrane was washed with PBS +0.02% Tween 80 (PBST) three times, each time for 5 min, added with horseradish peroxidase labelled rabbit anti‐mouse antibody (1:2000, Abcam), and incubated at room temperature for 2 h. Subsequently, the membrane was rinsed, added with ECL detection solution (Amersham Bioscience), and developed by conventional method X‐ray film. The relative protein bands were analysed by Scion Image Analysis System (Scion Corporation). The relative content of protein was expressed as the ratio of the OD of the target protein to β‐actin band.

### 2.11 Statistical analysis

Data were analysed with SPSS 22.0 (IBM Corp), and the measurement data are displayed in means ±standard deviation. The measurement data were with normal distribution. Independent *t*‐test or paired *t*‐test was employed for pairwise comparisons. One‐way analysis of variance (ANOVA) was employed for multiple group comparisons. Tukey's multiple comparisons test was used for the post hoc test. The correlation between the expression of CCAT2 and the efficacy of neoadjuvant chemotherapy was analysed by Kendall's tau‐b method. *p* < 0.05 was indicative of statistical significance.

## 3 RESULTS

### 3.1 BC cell sensitivity to 5‐Fu is negatively correlated with CCAT2 level

**FIGURE. 1:**
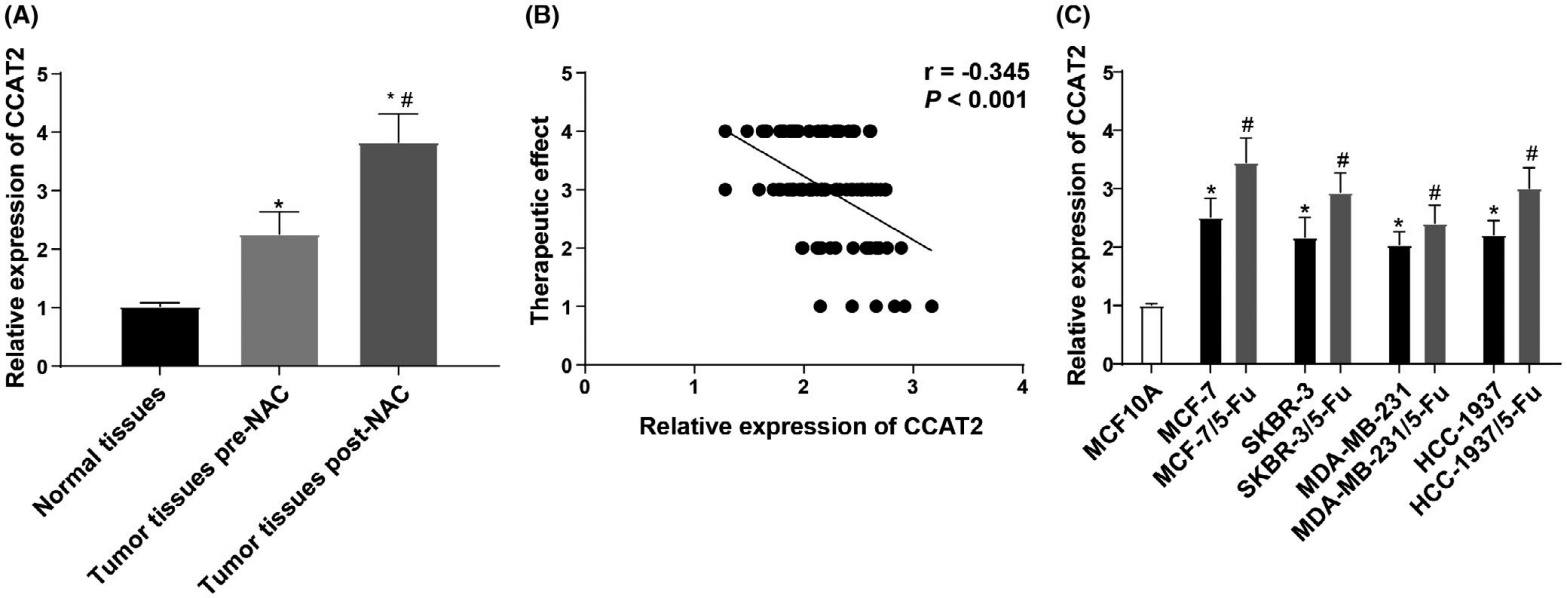
Sensitivity of BC to 5‐Fu is negatively correlated with the level of CCAT2. Note: A, expression of CCAT2 in normal breast tissues (n = 90) and BC tissues before (n = 100) and after neoadjuvant chemotherapy (n = 70) (PR +SD + PD); B, Kendall's tau‐b correlation analysis was used to detect the correlation between the effect of neoadjuvant chemotherapy and CCAT2 expression; C, expression of CCAT2 in BC cells and their corresponding drug‐resistant cells. The measurement data were expressed as mean ±standard deviation in panels A and C. For comparisons between two groups, independent *t*‐test was used for comparisons when compared to normal tissues group or MCF10A group. * *p* < 0.05; paired t‐test was used for comparisons when compared to tumour tissues pre‐neoadjuvant chemotherapy or corresponding BC non‐drug‐resistant cell lines, # *p* < 0.05. For comparisons among multi‐groups, one‐way ANOVA was used for comparisons. Tukey's multiple comparisons test was used for the post hoc test

Before and after chemotherapy, CCAT2 expression was detected by RT‐qPCR. It displayed that CCAT2 expression in BC tissues before neoadjuvant chemotherapy was higher than that in normal breast tissues. In addition, after chemotherapy, there were 30 cases of CR, 47 cases of PR, 17 of SD, and 6 of PD, with the total effective rate was 77.00%. Increased CCAT2 expression was found in residual tissues after chemotherapy (Figure 1A). Kendall's tau‐b correlation analysed the relationship between CCAT2 expression before chemotherapy and the efficacy of neoadjuvant chemotherapy. It claimed CCAT2 expression was negatively related to the chemotherapy efficacy in BC patients (*p* < 0.05) (Figure 1B). The lower the CCAT2 level, the better the efficacy of neoadjuvant chemotherapy in BC patients.

Compared with MCF10A cells, CCAT2 expression in BC cells was noticeably increased. MCF‐7 cells showed the highest expression of CCAT2, while MDA‐MB‐231 cells presented the lowest expression of CCAT2. Compared with parent BC cells, CCAT2 expression in 5‐Fu drug‐resistant cells was evidently increased (all *p* < 0.05) (Figure 1C), suggesting that CCAT2 may influence the drug sensitivity of BC cells to 5‐Fu.

### 3.2 mTOR pathway activities in drug‐resistant cells decreased after CCAT2 inhibition in BC

**FIGURE. 2:**
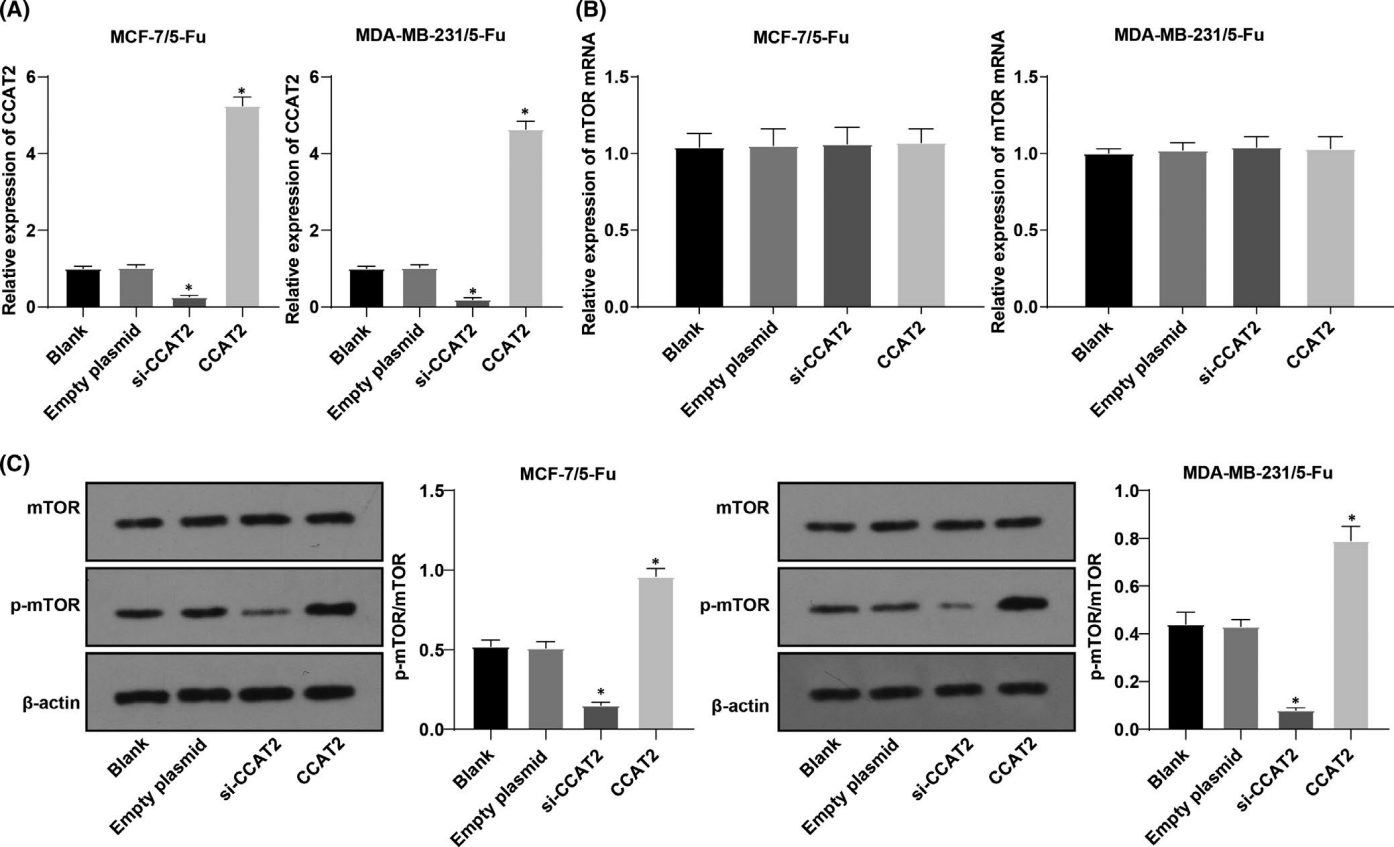
mTOR pathway activity in drug‐resistant cells decreased after CCAT2 inhibition in BC. Note: A, the mRNA level of CCAT2 was detected by RT‐qPCR; B, mRNA expression of mTOR in MCF‐7/5‐Fu cell and MDA‐MB‐231/5‐Fu cell detected by RT‐qPCR; C, Western blot analysis was used to detect expression of mTOR and p‐mTOR. The measurement data were expressed as mean ±standard deviation. One‐way ANOVA was used for comparisons. Tukey's multiple comparisons test was used for the post hoc test. Compared with the blank group, * *p* < 0.05

BC 5‐Fu‐resistant cells MCF‐7/5‐Fu and MDA‐MB‐231/5Fu were transfected with si‐CCAT2 or CCAT2 overexpression plasmid. The mRNA level of CCAT2 was detected by RT‐qPCR, which verified the transfection was successful (Figure 2A). The transcription level of mTOR (Figure 2B) and its phosphorylation (Figure 2C) were further detected by RT‐qPCR and Western blot. After the knockdown of CCAT2, the ratio of p‐mTOR/mTOR was decreased, while the ratio was increased after overexpression of CCAT2. It indicated that the mTOR pathway activity in drug‐resistant BC cells was decreased after CCAT2 inhibition.

### 3.3 Combination effects of CCAT2 and mTOR pathway on 5‐Fu drug resistance in BC

**FIGURE. 3:**
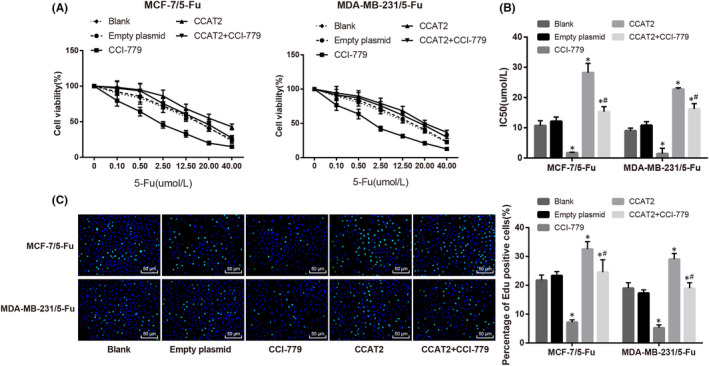
Combination effect of CCAT2 and mTOR signaling pathway on 5‐Fu drug resistance in BC. Note: A, cell growth curve; B, IC50 value in each group; C, EdU assay was performed to detect cell proliferation. The measurement data were expressed as mean ±standard deviation. Paired *t*‐test was used for comparisons between two groups and one‐way ANOVA was used for comparisons. Tukey's multiple comparisons test was used for the post hoc test. Compared with the empty plasmid group, * *p* < 0.05, compared with the CCI‐779 group, # *p* < 0.05

5‐Fu‐resistant cells were treated with CCI‐779. Versus the empty plasmid group, IC50 and cell proliferation activity were increased in the CCAT2 group, while decreased significantly in the CCI‐779 group (all *p* < 0.05). Versus MCF‐7/5‐Fu cells and MDA‐MB‐231/5‐Fu cells in the CCAT2 group, IC50 and cell proliferation activity in the CCAT2 + CCI‐779 group were noticeably decreased (all *p* < 0.05) (Figure 3). It showed that inhibition of the mTOR pathway attenuated the enhancement of CCAT2 on 5‐Fu drug resistance in BC.

### 3.4 Combination effects of CCAT2 and mTOR pathway on 5‐Fu drug‐resistant cell apoptosis in BC

**FIGURE. 4:**
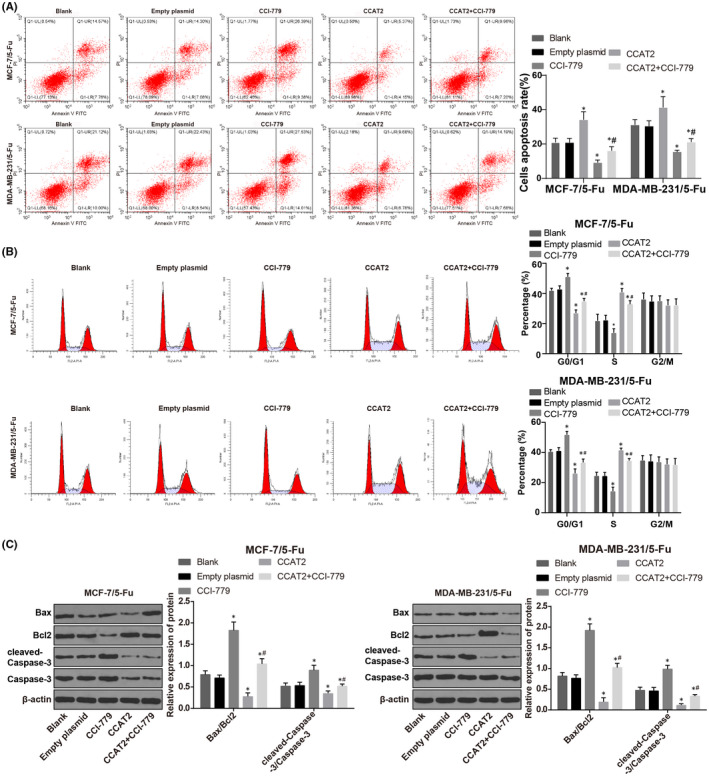
Combination effect of CCAT2 and mTOR pathway on 5‐Fu drug‐resistant cell apoptosis in BC. Note: A, flow cytometry was conducted to detect cell apoptosis; B, flow cytometry was applied to detect cell cycle; C, Western blot analysis was used to test apoptosis‐related factors. The measurement data were expressed as mean ±standard deviation. Paired *t*‐test was used for comparisons between two groups and one‐way ANOVA was used for comparisons. Tukey's multiple comparisons test was used for the post hoc test. Compared with the empty plasmid group, * *p* < 0.05, compared with the CCI‐779 group, # *p* < 0.05

The results showed that (Figure 4) compared with 5‐Fu‐resistant cells in the empty plasmid group, apoptosis rate, Bax/Bcl‐2, cleaved‐Caspase‐3/Caspase‐3, and G0/G1 cell proportion decreased, S cell proportion enhanced substantially in the CCAT2 group, while the CCI‐779 group showed opposite trends. Compared to the CCAT2 group, those indicators were markedly reduced in the CCAT2 + CCI‐779 group (all *p* < 0.05). Briefly, inhibition of the mTOR pathway reversed the anti‐apoptosis effect of CCAT2 on 5‐Fu drug‐resistant cells of BC.

### 3.5 CCAT2 mediates the BC occurrence and its sensitivity to 5‐Fu

**FIGURE. 5:**
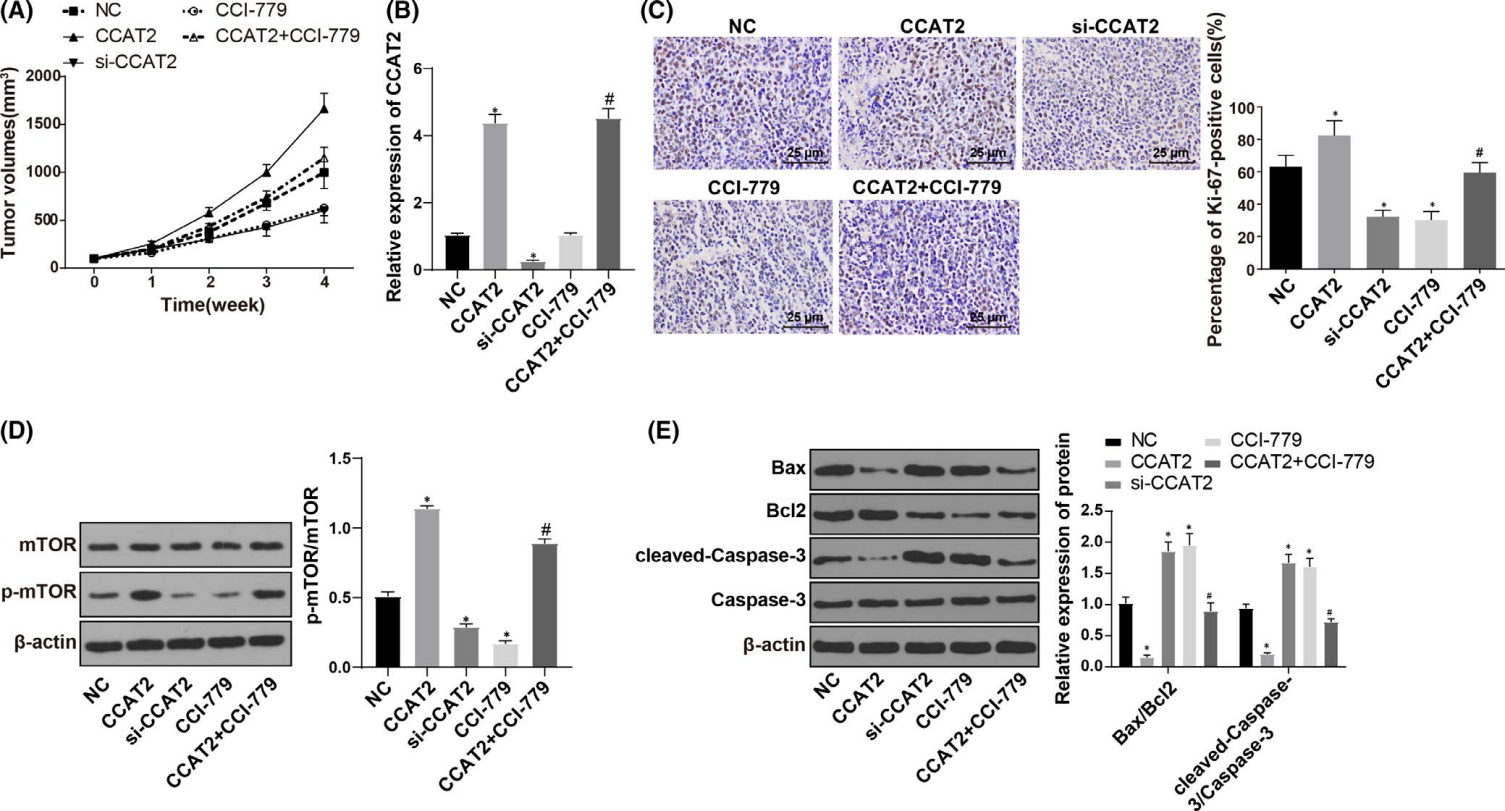
CCAT2 mediates the occurrence of BC and its sensitivity to 5‐Fu. Note: (A), changes of tumour volume in nude mice in each group; (B), the level of CCAT2 in transplanted tumour tissues in nude mice in each group; (C), immunohistochemistry was conducted to detect the Ki‐67 in xenografts of nude mice in each group (× 400) and positive expression rate of Ki‐67; (D), Western blot analysis was applied to detect expression of mTOR pathway‐related factors in transplanted tumour tissue; (E), Western blot analysis was used to test apoptosis‐related factors in transplanted tumour tissue. The measurement data were expressed as mean ±standard deviation. Paired *t*‐test was used for comparisons between two groups and one‐way ANOVA was used for comparisons. Tukey's multiple comparisons test was used for the post hoc test. Compared with the NC group, * *p* < 0.05, compared with the CCAT2 group, # *p* < 0.05

After the successful modelling, the volume of transplanted tumours gradually expanded. As is shown in Figure 5A, the growth rate of transplanted tumours was faster in the CCAT2 group than in the NC group; the growth rate of transplanted tumours in the si‐CCAT2 and CCI‐779 groups was slower. The volume of transplanted tumours in the CCAT2 + CCI‐779 group was less than that in the CCAT2 group (all *p* < 0.05). After removal of tumour in the nude mice, the mRNA level of CCAT2 was detected by RT‐qPCR. Compared with the NC group, the level of CCAT2 was clearly increased in the CCAT2 group and CCAT2 + CCI‐779 group and was clearly decreased in the si‐CCAT2 group (Figure 5B). The number of brownish yellow positive expressing tumour cells of proliferation marker protein Ki67 was detected by immunohistochemistry. The results revealed that (Figure 5C) relative to the NC group, the positive rate of Ki‐67 increased in the NC group; increased in the CCAT2 group; and decreased in the si‐CCAT2, CCI‐779, and CCAT2 + CCI‐779 groups versus the NC group, suggesting overexpression of CCAT2 *in vivo* could improve the drug resistance of 5‐Fu in BC‐resistant transplanted tumours. However, inhibition of mTOR reversed the effect of CCAT2 on 5‐Fu drug resistance in BC drug‐resistant xenografts. The mTOR pathway was detected by Western blot. Compared with the NC group, p‐mTOR/mTOR was decreased in the si‐CCAT2 group and CCI‐779 group and was increased in the CCAT2 group. Compared with the CCAT2 group, the phosphorylation level of mTOR was decreased in the CCAT2 + CCI‐779 group (all *p* < 0.05) (Figure 5D). Western blot analysis was also performed to detect the apoptosis‐related protein expression. Compared with the NC group, the levels of Bax/Bcl2 and cleaved‐Caspase‐3/Caspase‐3 ratio were diminished in the CCAT2 group; the si‐CCAT2 and CCI‐779 groups had increased levels; compared with the CCAT2 group, the levels of Bax/Bcl2 and cleaved‐Caspase‐3/Caspase‐3 were increased (all *p* < 0.05) (Figure 5E).

## 4 DISCUSSION

Although many efforts have been made in early diagnosis and treatment methods, BC survival rate keeps low due to poor prognosis.^23^ In addition, the process of BC treatment becomes more complex due to different therapeutic responses and outcomes.^24^ Therefore, with the expectation of providing a new insight for BC treatment, we enrolled 100 BC patients receiving neoadjuvant chemotherapy and conducted cell experiments and *in vivo* experiments to discuss the regulatory roles of CCAT2 in BC cell drug sensitivity to 5‐Fu via the mTOR pathway. Finally, we made a conclusion that CCAT2 may reduce BC cell chemosensitivity to 5‐Fu by activating the mTOR pathway.

CCAT2 expression before and after neoadjuvant chemotherapy was firstly detected. The results found that CCAT2 was overexpressed in BC, and the lower the CCAT2 expression, the better the efficacy of neoadjuvant chemotherapy. Increasing evidence manifested that abnormal lncRNA expression is implicated in tumourigenesis, and its close relationship with malignancy has attracted more and more attention recently.^25^, ^26^ Overexpression of CCAT2 was observed in many malignant tumours, and it may be a new oncogene.^13^, ^16^ CCAT2 is highly conserved and contains the SNP rs6983267, which was related to the risk of thyroid, colorectal, and prostate cancers.^27^, ^28^, ^29^ Consistently, Redis RS et al proved that significantly increased CCAT2 level was observed in BC tissues and could contribute to metastases‐free survival and longer overall survival for BC patients with lymph node positive receiving adjuvant CMF.^30^

Additionally, we observed that mTOR pathway activity in drug‐resistant cells decreased after CCAT2 inhibition in BC. CCAT2 was reported to induce chromosomal instability and metastases and modulate MYC expression, known to regulate kinds of axis controlling molecular process and cancer metabolism.^31^ mTOR is an evolutionarily conserved Ser/Thr protein kinase, and mTORC1 serves as a convergence point for PI3K/AKT to modulate cell activities.^32^ Besides, abnormal proliferation and anti‐apoptotic signals transmitted by the mTOR axis are involved in malignancy.^33^, ^34^ Further, activation of Akt, mTOR, and p70S6K has proved to be related to a more severe prognosis in BC patients.^35^ Cells with highly expressed Akt were more sensitive to mTOR inhibitors, while inhibiting mTOR might recover their sensitivity to chemotherapies.^35^, ^36^ Inhibitors targeting mTOR may suppress drug resistance.^37^ Previous evidence showed that CCAT2 knockout repressed endometrial cancer cell growth and invasion by binding to miR‐216b, and miR‐216b could negatively regulate Bcl‐2 that could active the mTOR pathway.^38^ Additionally, CCAT2 genetic polymorphisms were closely related to the susceptibility of lung cancer cells to platinum‐based chemotherapy response; thus, it may be an underlying biomarker for the disease prediction and chemotherapy response.^39^

Furthermore, inhibition of the mTOR pathway could reverse the anti‐apoptosis effect of CCAT2 on 5‐Fu drug‐resistant BC cells. Close interactions were found between apoptosis and autophagy via a common axis, among which the PI3K/AKT/mTOR is important in tumourigenesis.^40^ Functionally, CCAT2 is considered to be a therapeutic target because its depletion can block cancer cell proliferation and invasiveness.^41^ Besides, it is reported that higher CCAT2 expression is strongly linked with the more malignant molecular process in patients with gastric cancer (GC), and silencing CCAT2 prevented GC progression.^42^ Silencing CCAT2 triggered enhancement of GC cell apoptosis by suppression of PI3K and mTOR.^43^ Moreover, CCAT2 enhanced BC cell migration and decreased chemosensitivity to 5‐FU.^30^ In line with our study, silencing CCAT2 suppressed endometrial cancer cell growth by inactivation of the mTOR pathway.^38^ Finally, *in vivo* experiments found that overexpression of CCAT2 *in vivo* can increase the drug resistance to 5‐Fu in BC‐resistant transplanted tumour tissue, but inhibition of mTOR pathway can reverse the effect of CCAT2 on 5‐Fu resistance in BC‐resistant transplanted tumour tissue, which was consistent with our cell experiment results.

To summarize, CCAT2 was at a high level in BC tissues and cells, and CCAT2 may reduce chemosensitivity to 5‐Fu in BC cells by activating the mTOR pathway, manifesting CCAT2 may be a potential biomarker and therapeutic target for BC patients.

## CONFLICT OF INTEREST

The authors declare no potential conflicts of interest.

## AUTHOR CONTRIBUTION

**Daoping Zhou:** Conceptualization (equal); Project administration (equal); Writing‐original draft (equal); Writing‐review & editing (equal). **Juan Gu:** Conceptualization (equal); Project administration (equal); Writing‐original draft (equal); Writing‐review & editing (equal). **Yueping Wang:** Data curation (equal); Formal analysis (equal); Funding acquisition (equal); Validation (equal). **Bing Luo:** Data curation (equal); Formal analysis (equal); Funding acquisition (equal); Validation (equal). **Mei Feng:** Data curation (equal); Formal analysis (equal); Funding acquisition (equal); Supervision (equal). **Xuedong Wang:** Data curation (equal); Formal analysis (equal); Visualization (equal).

## Data Availability

The data that support the findings of this study are available from the corresponding author upon reasonable request.
