# Correction to “Long Noncoding RNA CCAT2 Reduces Chemosensitivity to 5‐Fluorouracil in Breast Cancer Cells by Activating the mTOR Axis”

**DOI:** 10.1111/jcmm.70238

**Published:** 2024-12-27

D. Zhou, J. Gu, Y. Wang, B. Luo, M. Feng, and X. Wang, “Long Noncoding RNA CCAT2 Reduces Chemosensitivity to 5‐Fluorouracil in Breast Cancer Cells by Activating the mTOR Axis,” *Journal of Cellular and Molecular Medicine* 26, no. 5 (2022): 1392–1401, https://doi.org/10.1111/jcmm.17041.

In Zhou et al. the results of the Kendall's tau‐b analysis in Figure 1B was mistakenly presented. The results should have been displayed in a table format and added as Table 2 to the article. The corrected Figure 1 and Table 2 are shown below. The authors confirm all results, and conclusions of this article remain unchanged.

**TABLE 2 |** The correlation of neoadjuvant chemotherapy efficacy with CCAT2 expression level was analysed by Kendall's tau‐b correlation analysis.

**Table:** 

	Parameters	Efficacy of neoadjuvant chemotherapy
CCAT2 expression level	Correlation index	0.345
	*p*	< 0.001


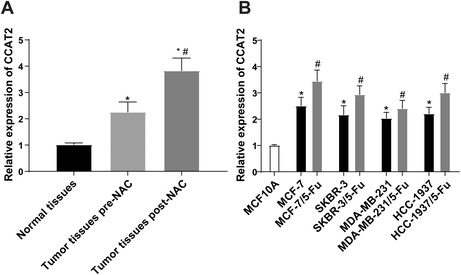
 **FIGURE 1 |** The sensitivity of BC to 5‐Fu is negatively correlated with the level of CCAT2. (A) Expression of CCAT2 in normal breast tissues (*n* = 90) and BC tissues before (*n* = 100) and after neoadjuvant chemotherapy (*n* = 70) (PR + SD + PD); (B) expression of CCAT2 in BC cells and their corresponding drug‐resistant cells. The measurement data were expressed as mean ± standard deviation in panels A and B. For comparisons between two groups, independent *t*‐test was used for comparisons when compared to normal tissues group or MCF10A group. *p < 0.05; paired *t*‐test was used for comparisons when compared to tumour tissues pre‐neoadjuvant chemotherapy or corresponding BC non‐drug‐resistant cell lines, #*p* < 0.05. For comparisons among multi‐groups, one‐way ANOVA was used for comparisons. Tukey's multiple comparisons test was used for the post hoc test.

We apologize for these errors.

